# Epidermolysis Bullosa in children: the central role of the pediatrician

**DOI:** 10.1186/s13023-021-02144-1

**Published:** 2022-04-04

**Authors:** Maria Rosaria Marchili, Giulia Spina, Marco Roversi, Cristina Mascolo, Elisabetta Pentimalli, Marialuisa Corbeddu, Andrea Diociaiuti, Maya El Hachem, Alberto Villani

**Affiliations:** 1 Department of Emergency, Acceptance and General Pediatrics, Bambino Gesù Children’s Hospital, IRCCS, 00165 Rome, Italy; 2 Dermatology Unit and Genodermatosis Unit, Genetics and Rare Diseases Research Division, Bambino Gesù Children’s Hospital, Istituti di Ricovero e Cura a carattere Scientifico, Rome, Italy; 3 Postgraduate School of Paediatrics, University of Torino, Turin, Italy

**Keywords:** Epidermolysis bullosa, Complications, Multidisciplinary approach, Nutritional impairment, Pediatric primary healthcare

## Abstract

Epidermolysis bullosa (EB) is a severe hereditary disease characterized by defective epithelial adhesion causing mucocutaneous fragility. The major types are EB simplex (EBS), junctional EB (JEB), dystrophic EB (DEB) and more than 35 EB subtypes. Another very rare type of EB is Kindler EB (KEB). Clinically, it is a very heterogeneous disease which ranges from localized to extensive skin lesions with frequent multisystem extra cutaneous involvement. The role of a pediatrician-dermatologist cooperation within a multidisciplinary team is fundamental for both the diagnosis and management contributing to these patients’ better life expectancy. Aim of this study is to describe clinical and laboratory characteristics of the main EB subtypes focusing on nutritional and gastrointestinal aspects, providing information to aid the paediatric management of children with EB. This retrospective study reviewed the cases of 160 pediatric EB patients (76 male and 84 female): 31 patients affected by EBS (mean age ± SD: 4.37 ± 7.14), 21 patients affected by JEB (mean age ± SD: 9.26± 17.30) and 108 with DEB (mean age ± SD: 11.61 ± 13.48). All patients were admitted at the Bambino Gesù Children’s Hospital in Rome, between June 2005 to June 2020. The reduced gastrointestinal absorption, chronic losses, esophageal stenosis and chronic inflammatory state, represent the basis of nutritional problems of EB patients. In particular, anemia represents one of the most important complications of DEB patients which could require transfusion-dependent patterns. Malnutrition, vitamin deficiencies and anemia have been related to growth delay in EB patients. A specific diet with a balance of all macronutrients is required and improving caloric intake with sugar limitations is fundamental to prevent dental caries and tooth decay typical of EB patients. While sepsis proved to be the major cause of morbidity and mortality in younger patients, squamous cell carcinoma was mostly observed in older patients, especially those affected by DEB. Patients with EB require regular monitoring for complications and sequelae with a frequency of evaluations which varies based on age and EB subtypes. Cooperation among medical teams involving paediatricians, dermatologists, specialist clinicians including nutritionists such as families and patient’s association is fundamental to approach the disease and improve the quality of life of these patients.

**Supplementary Information:**

The online version contains supplementary material available at 10.1186/s13023-021-02144-1.

## Background

Inherited epidermolysis bullosa (EB) is a heterogeneous group of genodermatoses characterized by defective epithelial adhesion causing mucocutaneous fragility with blister formation after minimal trauma [1, 2]. EB classification has been recently revised confirming 4 major types: EB simplex (EBS), junctional EB (JEB), dystrophic EB (DEB) and more than 35 EB subtypes [1]; moreover, a very rare type of EB is Kindler EB (KEB) [1]; To date, the involved genes are 16 coding for 13 proteins [1]. Inheritance modality is autosomal recessive or dominant depending on EB subtype such as dominant DEB (DDEB), severe recessive DEB (RDEB) [1].

Incidence and prevalence of EB have been widely and variously defined through epidemiological studies considering clinical or molecular characteristics. Variegated data have been reported reflecting variations in patient’s recruitment from US to Europe [3, 4]; in more detail: EBS is the most frequent type followed by DEB which incidence was reported as 6 per million life births in USA and Spain, with the highest value in Scotland (20 per million) [1].

EB clinical spectrum ranges from localized to extensive skin lesions with frequent extensive multisystem extra cutaneous involvement [1, 3, 4]. Life expectancy can be normal or variably reduced, including early lethal forms especially in RDEB and in generalized junctional EB because of sepsis, respiratory failure or squamous cell carcinoma (SCC) [5].

EB recognition and early diagnosis is necessary: the role of the pediatricians, especially those involved in pediatric primary healthcare evaluation, is fundamental for both the diagnosis and management of EB children to contribute to these patients’ better life expectancy. In this scenario, pediatrician and dermatologist cooperation is required. This aspect is under consideration in all European Countries. Indeed, the European Reference Network for Rare Skin Disorders (ERN-Skin) is focusing a major part of the activities to spread the knowledge on EB in non-reference centers [6].

The aim of this study is to describe the clinical and laboratory characteristics of the three main EB subtypes and to provide recommendations to aid the pediatric management of children with EB. This would help define the role of the pediatrician-dermatologist cooperation within the multidisciplinary team composed of professionals such as dentists, pain therapist, physiotherapist, psychologists, endocrinologist, endoscopist and nutritionists as well as families and patients associations.

## Methods

We retrospectively reviewed the cases of 160 patients of EB in children admitted at the Bambino Gesù Children’s Hospital in Rome, between June 2005 to June 2020. The Dermatology Unit in Bambino Gesù Children’s Hospital is a reference center for rare skin disorders including EB and the dermatologists are the case manager of these patients. All patients are followed by a multidisciplinary team upon the patients’ needs. However, the dermatologists and the pediatricians are regularly consulting together all patients.

This retrospective study has been carried out within the framework of the European Reference Network for rare skin disorders (ERN-Skin) [6].

The diagnosis of EB was initially clinical and subsequently confirmed by immunofluorescence antigen mapping and ultrastructural examination of skin biopsies [7]. In the majority of the cases, a genetic study was performed to make a prediction on the clinical course according to the known gene mutations.

All data were deposited in a web dataset upon the first evaluation of the patient, either during hospitalization or day hospital recovery, depending on the severity of the disease. In particular, follow-up evaluation has been performed for all the duration of the disease along life.

For each patient, we reported the following clinical and laboratory variables (when available), classified both per age group (0 to 1 year, 1.01 to 10 years, 10.01 to 20 years, >20 years) and main EB subtypes (Simplex; Junctional; Dystrophic): age; age group; gender; percentile of BMI or W/H ratio; gene mutations; EB complications, including cutaneous, ocular, gastrointestinal, neuropsychiatric and other complications; laboratory workup, including indices of anemia, inflammation, nutritional state, renal function, liver function, coagulation, hormones, and others (see Tables 1, 2 and 3 and supplementary data for further details). In detail, the percentiles of BMI and weight-for-height (W/H) ratio were evaluated in patients older than 2 years old and younger than 2 years old, respectively, according to Italian growth charts and percentiles for boys and girls. Malnutrition was defined as follows: BMI <3° centile in patients from 2 to 20 years old, BMI <18.5 in patients older than 20 years old, W/H ratio <3° in patients younger than 2 years old [8].

**Table 1:** Demographic and genetic characteristics of EB patients

EB major types	EBS	JEB (all)	JEB (severe)	DEB
Total	**31**	**21**	**8**	**108**
Male, no. (%)	**17** (54.8)	**9** (42.9)	**2** (25)	**50** (46.3)
Female, no. (%)	**14** (45.2)	**12** (57.1)	**6** (75)	**58** (53.7)
**Age**				
Median, years (range)	0.88 (0–26)	0.09 (0–41.23)	0.08 (0–41.23)	4.67 (0–43.33)
Mean ± SD, years	4.37 ± 7.14	9.26 ± 17.30	9.93 ± 18.26	11.61 ± 13.48
**Follow-up**				
Median, years (range)	**1.35** (0–19.0)	0 (0–6.6)	0 (0–3,9)	1.39 (0 - 21.9)
Mean ± SD, years	3.67 ± 4.76	1.25 ± 2.15	0.98 ± 1.81	3.22 ± 4.49
**BMI or W/H percentiles**				
<3°	4/29 (14)	3/29 (10%)	1/3 (34 of all JEB)	22/29 (76)
3-10°	4/32 (13)	0/32 (0%)	0/0 (0 of all JEB)	28/32 (87)
>10°	19/75 (25)	14/75 (19%)	5/14 (36 of all JEB)	42/75 (56)
**Mutated genes**				
KRT14, n (%)	**5** (16.1)	0	0	0
KRT5, n (%)	**2** (6.5)	0	0	0
KLHL24, n (%)	**2** (6.5)	0	0	0
EXPH5, n (%)	**1** (3.2)	0	0	0
KRT5, n (%)	**1** (3.2)	0	0	0
LAMA3, n (%)	0	**3** (13%)	**3** (37.5)	0
LAMB3, n (%)	0	**2** (8.7%)	**1** (20)	0
LAMC2, n (%)	0	**1** (4.3%)	**1** (20)	0
*COL*17A1, n (%)	0	**3** (13%)	0	0
ITGB4, n (%)	0	**1** (4.3%)	0	0
COL7A1, n (%)	0	0	0	**27** (25)
PLEC, n (%)	**2** (6.5%)	0	0	0

Bold denotes either absolute numbers or significant *p*-values

**Table 2:** EB complications per EB major types

EB major types	All	EBS	JEB (all)	JEB (severe)	DEB	p-value*
Total	**160**	**31**	**21**	**8**	**108**	
**Skin complications**						
Alopecia, no. (%)	**4** (2.5)	**1** (1.6)	**1** (4.7)	**0** (0)	**3** (2.7)	0.891
Ankyloglossia, no. (%)	**20** (12.5)	**0** (0)	**0** (0)	**0** (0)	**20** (18.5)	**0.004**
Phimosis, no. (%)	**2** (1.3)	**0** (0)	**0** (0)	**0** (0)	**2** (1.9)	0.614
Microstomia, no. (%)	**8** (5)	**1** (1.6)	**2** (9.5)	**2** (25)	**5** (4.6)	0.565
Onychodystrophy, no. (%)	**48** (30)	**10** (32.3)	**10** (47.6)	**4** (50)	**28** (25.9)	0.133
Skin infections, no. (%)	**152** (95)	**27** (87.1)	**21** (100)	**1** (20)	**104** (96.3)	0.100
Squamous cell carcinoma, no. (%)	**16** (10)	**0** (0)	**0** (0)	**0** (0)	**16** (14.8)	**0.010**
Syndactyly, no. (%)	**32** (20)	**0** (0)	**0** (0)	**0** (0)	**32** (29.6)	**<0.001**
**Ocular complications**						
Corneal lesions, no. (%)	**8** (5)	**0** (0)	**0** (0)	**0** (0)	**8** (13.3)	0.132
Strabismus, no. (%)	**1** (0.6)	**1** (3.2)	**0** (0)	**0** (0)	0	0.123
Synechiae, no. (%)	**1** (0.6)	**0** (0)	**0** (0)	**0** (0)	**1** (0.9)	0.785
**Gastrointestinal complications**						
Constipation, no. (%)	**34** (21.3)	**2** (6.4)	**1** (4.8)	**0** (0)	**31** (28.7)	**0.004**
Esophageal stenosis, no. (%)	**55** (34.3)	**1** (3.2)	**1** (4.7)	**0** (0)	**53** (49.1)	**<0.001**
GERD, no. (%)	**2** (1.3)	**0** (0)	**0** (0)	**0** (0)	**2** (1.9)	0.614
Malnutrition, no. (%)	**80** (50)	**12** (38.7)	**4** (19.0)	**2** (25)	**64** (59.2)	**0.001**
Oral mucosal lesions, no. (%)	**17** (10.6)	**1** (3.2)	**4** (19.0) **2** (25)	**2** (25)	**12** (11.1)	0.184
Tooth decay, no. (%)	**68** (42.5)	**9** (29)	**6** (28.5%)		**53** (49)	0.053
**Neuropsychiatric disorders**						
Cognitive delay or ADHD, no (%)	**5** (3.1)	**2** (6.5)	**0** (0)	**0** (0)	**3** (2.8)	0.396
Depression, no (%)	**2** (1.3)	**0** (0)	**1** (4.7)		**1** (0.9)	0.275
Loss of ambulation, no (%)	**5** (3.1)	**0** (0)	**1** (4.7)	**0** (0)	**4** (3.7)	0.521
Sleep disorder, no. (%)	**2** (1.3)	**1** (3.2)	**0** (0)	**0** (0)	**1** (0.9)	0.512
**Other complications**						
Dysphonia, no. (%)	**3** (1.9)	**1** (3.2)	**1** (4.7)	**1** (20)	**1** (0.9)	0.409
Sepsis, no. (%)	**7** (4.4)	**0** (0)	**6** (28.5)	**1** (20)	**1** (0.9)	**<0.001**

Bold denotes either absolute numbers or significant *p*-values

*Calculated with the Chi-squared test

**Table 3:** Laboratory workup of EB patients (all ages)

	EBS	JEB	DEB	p-value*
*Anemia*				
Haemoblobin (g/dl)	12.6 (9.2–14.9) ± 1.3	10.9 (5.5–17.4) ± 3.5	10.4 (5.3–14.3) ± 1.9	**0.008**
Reticulocytes (%)	0.6 (0.4–1.4) ± 0.6	1.4 (0.8–2.5) ± 0.6	1.7 (0.8–12.8) ± 2.6	**0.046**
Serum iron (mcg/dl)	56 (18–109) ± 27	43 (12–48) ± 17	19.5 (5–477) ± 68	**0.005**
Ferritin (ng/ml)	29 (18–74) ± 19	14 (8–48) ± 16	22 (4–1039) ± 210	0.463
Hyperferritinemia no. (%)	0	0	4 (3.7%)	0.754
Transferrin (mg/dl)	271 (244–315) ± 28	270 (215–287) ± 29	234 (92–421) ± 73	0.431
*Inflammation*				
White blood cells (cells/mm3 *10^3^)	9.02 (4.54–11.83) ± 2.31	10.2 (8.74–13.81) ± 2.11	10.01 (3.95–16.67) ± 3.03	0.350
Neutrophils (%)	44 (35–73) ± 13	64 (51–66) ± 6.9	58.3 (1.5–81.3) ± 18.1	0.186
CRP (mg/dl)	0.15 (0.05–3.05) ± 1.19	2.13 (2.03–4.15) ± 1.19	3.2 (0.05–15.81) ± 4.23	**0.011**
ESR (mm/h)	8 (6–26) ± 8.3	35 (14–119) ± 56	36 (5–90) ± 25	**0.027**
IgA (mg/dl)	166 (66–214) ± 71.8	431 (156–988) ± 325	316 (5–831) ± 214	0.076
IgG (mg/dl)	1013 (731–1550) ± 344	1313 (1104–1766) ± 325	2056 (616–6564) ± 1484	**0.012**
IgM (mg/dl)	81 (42–128) ± 35	93 (70–127) ± 21	127 (37–491) ± 87	0.072
*Nutritional state*				
Glicemia (mg/dl)	81 (78–98) ± 6.9	88 (57–97) ± 16	84 (68–108) ± 10.5	0.770
Blood protein (g/dl)	6.8 (6.2–8) ± 0.6	7.3 (7–7.7)	7.7 (6.4–10.8) ± 0.99	**0.043**
Albumin (g/dl)	4.4 (4.1–4.7) ± 0.2	4.1 (3.4–4.3) ± 0.4	3.8 (2.07–4.6) ± 0.57	**0.011**
Pre-albumin (mg/dl)	17 (15–17) ± 1.2	17 (15–23) ± 4.2	12 (1.1–20) ± 4.3	**0.013**
RBP (mg/dl)	2.3 (1.7–2.6) ± 0.5	3.6 (2.39–3.6) ± 0.69	1.86 (1.17–2.88) ± 0.47	**0.001**
Total cholesterol (mg/dl)	138 (119–182) ± 24	168 (122–187) ± 24	129 (67–216) ± 27	**0.040**
Triglycerides (mg/dl)	49 (40–73) ± 12	80 (79–117) ± 20	73 (41–235) ± 38	**0.004**
Vitamin A (µmol/l)	1.3 (1.22–1.94) ±0.39	1.3 (1–3.9) ± 1.6	1.1 (0.4–2.7) ± 0.59	0.121
Vitamin D (ng/ml)	25 (19–31.1) ± 5.8	19.5 (10.8–27.2) ± 7.4	14.2 (3.7–60) ± 14.2	0.418
Vitamin E (µmol/l)	51.2 (33.3–53.9) ± 11.2	36.6 (29.2–100.5) ± 39.2	57.4 (24.9–105.3) ± 21.9	0.833
Vitamin B12 (pg/ml)	632 (452–1225) ± 404	457 (261–545) ± 145	654 (173–1586) ± 337	0.588
Folati (ng/ml)	6 (4–7.2) ± 1.6	7.4 (6–20.9) ± 8.3	7 (2.2–58) ± 14.8	0.395
Vitamin B6 (µmol/l)	60.5 (41.1–69.8) ± 14.6	15 (15–31) ± 9.2	39.9 (10–212) ± 52	0.252
*Renal function*				
Azotemia (mg/dl)	14 (12–17) ± 1.7	12 (11–33) ± 9.8	14 (12–17) ± 1.7	**0.006**
Creatinine (mg/dl)	0.47 (0.22–0.7) ± 0.16	0.62 (0.19–0.7) ± 0.21	0.35 (0.15–1.28) ± 0.23	0.538
Uric acid (mg/dl)	3.6 (2.5–3.9) ± 0.57	4.2 (3.9–19) ± 6.6	3.6 (2.5–3.9) ± 0.57	0.065
*Liver function*				
AST (IU/l)	28 (24–39) ± 5.4	24 (14–33) ± 7.7	22 (10–90) ± 12	0.062
ALT (IU/l)	15 (13–25) ± 4.7	10 (8–25) ± 8	10 (5–46) ± 7.6	0.061
GGT (IU/l)	8.5 (6–14) ± 2.7	12 (11–14) ± 1.5	8 (4–915) ± 137	0.500
Bilirubin (mg/dl)	0.41 (0.26–0.5) ± 0.09	0.4 (0.19–1.27) ± 0.43	0.25 (0.14–0.74) ± 0.14	**0.017**
Alkaline phosphatase (U/L)	428 (381–454) ± 31	171 (61–497) ± 178	282 (98–639) ± 147	**0.063**
*Coagulation*				
Platelets (cells/mm3*10^3^)	284 (202–327) ± 45	291 (241–486) ± 99	452 (55–1183) ± 188	**0.014**
Fibrinogen (mg/dl)	303 (239–366) ± 64	462 (368–511) ± 72.7	466 (275–731) ± 109	0.099
PT (s)	13.3 (12.4–13.5) ± 0.59	10 (10–12.9) ± 1.67	13.9 (12.1–17.6) ± 1.23	**0.003**
aPTT (s)	31.3 (26.8–32.0) ± 2.82	29.8 (28.9–30.3) ± 0.71	35.2 (27.8–45.7) ± 4.22	**0.008**
*Electrolytes*				
Sodium (mmol/l)	139 (136–143) ± 2.4	139 (136–141) ± 1.9	138 (130–143) ± 2.8	0.298
Potassium (mmol/l)	4.6 (4.07–4.94) ± 0.28	4.6 (4.2–4.7) ± 0.18	4.39 (3.5–5.4) ± 0.41	0.579
Chlorine (mmol/l)	106 (101–108) ± 2.7	103 (100–106) ± 4.2	103 (98–111) ± 2.9	0.278
Calcium (mg/dl)	9.8 (9.2–10) ± 0.3	9.3 (8.8–9.7) ± 0.38	9.2 (2.08–10) ± 1.17	**0.034**
Magnesium (mg/dl)	2.18 (2.02–2.22) ± 0.08	2 (1.85–2.07) ± 0.08	2.1 (0.75–3.94) ± 0.4	0.122
Phosphate (mg/dl)	5.05 (4.4–5.3) ± 0.39	3.6 (3.3–5.4) ± 0.98	4.55 (1.17–6.3) ± 0.89	0.176
*Hormones*				
PTH (pg/ml)	25 (21–29) ± 4	54 (36–63) ± 13.7	30 (8 -1285) ± 233	0.179
TSH (U/ml)	2.85 (2.51–3.84) ± 0.59	1.22 (0.91–2.3) ± 0.73	2.03 (0.72–5.12) ± 1.25	0.138
FT4 (ng/dl)	1.16 (1.07–1.23) ± 0.08	1.31 (1.22–2.69) ± 0.82	1.12 (0.79–1.66) ± 0.21	0.125
*Other*				
Amylase (U/L)	66 (56–96) ± 20.8	55 (30 -139) ± 43	64 (24–670) ± 113	0.861
Copper (µr/dl)	82 (9–95) ± 8.3	96 (88–169) ± 38.3	107 (9.7–156) ± 32	0.521

Bold denotes either absolute numbers or significant *p*-values

*Calculated with the One-Way ANOVA test for normally distributed variables and the Kruskal-Wallis test for non-normally distributed variables

Anemia was defined considering age related differences and percentile curves for hemoglobin (Hb) as following: Hb <14.5 g/dl in new-borns; Hb <10 g/dl in patients from 1 to 2 months; Hb <9 g/dl in patients from 2 to 3 months; Hb <9.5 g/dl in patients from 3 to 6 months; Hb <11 g/dl in patients from 6 to 12 months; Hb <11.5 g/dl in patients from 2 years to 12 years; Hb <12 g/dl in female older than 12 years 86 old and Hb <13 g/dl in males older than 12 years old [9]. Cut-off points to define Vitamin D status are: Vitamin D insufficiency if values range88 from 20 to 29 ng/ml; vitamin D deficiency in case of value lower than 20 ng/ml; vitamin D severe deficiency in case of values lower than 10 ng/ml [10]. Vitamin A, Vitamin B6, Vitamin B12 and folate low limit values were defined considering the Bambino Gesù Children’s Hospital laboratory normal range.

The software IBM SPSS version 23.0 was used for statistical analysis. All continuous data were expressed as means and standard deviations or as medians and ranges if normally and non-normally distributed, respectively. All categorical variables were expressed as proportions and percentages. Data were reported partitioned per the age group or EB subtype or both. Subgroup analysis was performed with the ANOVA test for normally distributed continuous variables, the Kruskal-Wallis test for non-normally distributed continuous variables and the Chi-squared test for categorical variables. Multivariate analysis was performed via ordinal logistic regression, adopting the percentiles of BMI as the dependent variable and significant variables from univariate analysis as the independent variables, whose choice is discussed later in the text. A p-value less than 0.05 was considered statistically significant.

## Results

The demographic and genetic characteristics of the studied sample are outlined in Table 1.

During the study period, a total of 160 patients with epidermolysis bullosa were referred to the Bambino Gesù Children’s Hospital. The distribution of EB types and subtypes, according to the latest classification [1], was: EBS: 31 patients (19.4%); JEB: 21 patients (13.1%); DEB: 108 patients (67.5%). Kindler patients have not been considered because follow-up has been missed because of geographical provenience of these patients.

The mean age at diagnosis was 36 months (data not shown). A genetic mutation has been documented in a percentage of 26.95% with parents being the most affected followed by siblings, cousins and grandparents. Moreover, a percentage of 9.6% of our sample were blood relatives (data not shown).

The organ-specific complications occurring in the three EB major types are outlined in Table 2.

The laboratory workup of EB patients per EB major types and age group are outlined in Tables 1 and 3 of Additional file 1.

The normal ranges of the reported laboratory parameters are shown in Table 4.

**Table 4:** Normal ranges of laboratory parameters

	Normal range		Normal range
*Anemia*		***Renal function***	
Haemoblobin (g/dl)	10.5–15.5	Azotemia (mg/dl)	5–18
Reticulocytes (%)	0.2–2	Creatinine (mg/dl)	0.44–0.68
Serum iron (mcg/dl)	35–130	***Liver function***	
Ferritin (ng/ml)	9–290	AST (IU/l)	<40
Transferrin (mg/dl)	240–360	ALT (IU/l)	<41
*Inflammation*		GGT (IU/l)	<60
White blood cells (cells/mm3 * 10^3^)	4–11 *10^3^	Bilirubin (mg/dl)	
Neutrophils (%)	33–59	Alkaline phosphatase	140–400
CRP (mg/dl)	0–0.5	***Coagulation***	
ESR (mm/h)	0–15	Platelets (cells/mm3*10^3^)	150–400
IgA (mg/dl)	70–400	Fibrinogen (mg/dl)	212–433
IgG (mg/dl)	700–1600	PT (s)	16–22
IgM (mg/dl)	40–230	aPTT (s)	28–37.9
*Nutritional state*		***Electrolytes***	
Glicemia (mg/dl)	60–100	Sodium (mmol/l)	136–145
Blood protein (g/dl)	6.4–8.3	Potassium (mmol/l)	3.1–5.1
Albumin (g/dl)	3.5–5.5	Chlorine (mmol/l)	98–107
Pre-albumin (mg/dl)	15–30	Calcium (mg/dl)	8.8–10.8
RBP (mg/dl)	3.5–5	Magnesium (mg/dl)	1.7–2.1
Total cholesterol (mg/dl)	105–223	Phosphate (mg/dl)	3.2–5.7
Triglycerides (mg/dl)	<170	***Hormones***	
Vitamin A (µmol/l)	0.91–1.71	PTH (pg/ml)	15–65
Vitamin D (ng/ml)	20–120	TSH (U/ml)	0.51–4.3
Vitamin E (µmol/l)	7–21	FT4 (ng/dl)	0.98–1.64
Vitamin B12 (pg/ml)	197–711	***Other***	
Folati (ng/ml)	8.60–37.70	Amylase (U/L)	<31
Vitamin B6 (µmol/l)	17.4–70.8	Copper (mcr/dl)	75–145
Uric acid (mg/dl)	3.4–7		

### Skin infections

Skin infections were described in a percentage of 95% of the total cohort: DEB patients were the most affected (87%), followed by JEB (13.1%) and EBS (16.9%). All infections were caused by S. Aureus (data not shown). A total of 4.4% of patients had S. Aureus sepsis (6 with JEB and 1 with DEB). Ankyloglossia, onychodystrophy, syndactyly and alopecia were mostly detected in DEB patients. Squamous cell carcinoma was found in a percentage of 10% of the entire population. All of these patients had DEB, 41.2% died of this complication, and 23.5% required amputations, respectively (data not shown). Severe skin itch was detected in one DEB patient (data not shown).

### Gastrointestinal complications

Esophageal stenosis was documented in a percentage of 34.4%: DEB (33.1%), JEB (0.6%), EBS (0.6%). Of those, a percentage of 81.4% received Esophageal dilatation. Constipation was observed in a percentage of 21.2%, the majority affected by DEB (19.4%). Tooth decay was observed in a percentage of 42.5% of the total sample. Celiac disease was found in 1 JEB patient (data not shown).

### Malnutrition and growth delay

Malnutrition was detected in a percentage of 50% of the total sample. Patients with DEB patients were the most affected with a percentage of 59.2%. A BMI lower than the 3rd centile was found in a percentage of 9.4% in the group of patients aged less than 1 year. Moreover, a weight/length ratio lower than the 3rd centile was documented in a percentage of 10% in the group of patients younger than 2 years old (data not shown). Only 2.9% of patients (1 patient per each age group) had a BMI above the 97th centile or higher than the reference values or a weight for length ratio above than the 97th centile.

The BMI percentiles per age group are shown in Table 5.

**Table 5:** BMI centiles in EB patients per age group

	<3°	3-10°	>10°	Total
0–1 yr	**13** (9.4%)	**20** (14.4%)	**34** (24.5%)	**67** (48.2%)
1–10 yr	**9** (6.5%)	**5** (3.6%)	**12** (8.6%)	**26** (18.7%)
10–20 yr	**4** (2.9%)	**2** (1.4%)	**10** (7.2%)	**16** (11.5%)
>20 yr	**3** (2.2%)	**6** (4.3%)	**21** (15.1)	**30** (21.6%)
Total	**29** (21%)	**33** (23.7%)	**77** (55.4%)	**139**

Bold denotes either absolute numbers or significant *p*-values

A deficit of total protein and serum albumin was also detected, as shown in Tables 1 and 3 of Additional file 1.

To evaluate the predictors of the BMI change in EB patients, we performed an ordinal logistic regression adopting the percentiles of BMI (<3, 3 to 10, >10°) as the dependent variables. As shown in Table 6, we found a significant association between the increase of serum albumin and the decrease of feeding problems with the increase of the correspondent BMI range.

**Table 6:** Ordinal logistic regression (dependent variable: percentiles of BMI and W/H ratio)

	OR	*p* value
Haemoblobin (g/dl)	0.018	0.739
Albumin (g/dl)	3.156	**0.015**
IgG (mg/dl)	0.001	0.282
Feeding problems	− 2.396	**0.013**

Bold denotes either absolute numbers or significant *p*-values

### Anemia

Anemia was detected in 66 patients (41.3%), 51 of whom were affected by 148 DEB (77.2%). The most affected patients were aged less than 1 years (46.9%), followed by those older than 20 years (31.8%).

The mean values of serum iron, ferritin, and transferrin are shown in Tables 1 and 3 of Additional file 1.

The number of anemic patients per age group and EB subtypes is shown in Table 7.

**Table 7:** Frequency of blood exams and investigations in patients with severe EB major types

Investigations	0–1 y patients	1–10 y patients	10–20 y patients	>20 y patients
Blood exams^1^	6–12 months^2^	12 months	12 months	12 months
Hormonal assessment	–	–	12 months	12 months
Specialist evaluation^3^	6–12 months	6–12 months	6–12 months	6–12 months
Psychological evaluation	6–12 months (parents)	6–12 months (Parents and child)	6–12 months (Parents and child)	6–12 months (Parents and child)
Imaging examinations^4^	12 months	12 months	12 months	12 months
Dual X-ray	–	–	12 months	12 months

Vaccinations should be performed for all EB patients independently on age and EB types, according with Vaccination Action Plan

^1^Complete blood cell count, hemoglobin, mean cellular volume, reticulocytes, ferritin, serum iron, CRP, ESR, renal function, electrolytes, immunoglobulin, total protein, albumin, zinc, selenium, vitamin D, B6, folate, vitamin B12, carnitine

^2^To be anticipated in case of acute signs or symptoms of the disease manifesting before the given age

^3^Dentist, pain therapist, physiotherapist, endocrinologist, endoscopist, hematologist, nutritional experts, ophthalmologist, cardiologist in addition to pediatricians and dermatologists.

^4^Orthopantomograph

**Table 8:** Anemia in EB patients per age group and EB major types

	EBS	JEB	DEB	Total
0–1 yr	**5** (7.5%)	**8** (12%)	**18** (27%)	**31** (46.9%)
1–10 yr	**0** (0%)	**0** (0%)	**8** (12%)	**8** (12%)
10–20 yr	**0** (0%)	**0** (0%)	**6** (9.1%)	**6** (9.1%)
>20 yr	**0** (0%)	**2** (3%)	**19** (29%)	**21** (31.8%)
Total	**5** (7.6%)	**10** (15.1%)	**51** (77.2%)	**66**

Bold denotes either absolute numbers or significant *p*-values

### Vitamin deficiency

Vitamin D deficiency was mostly observed in patients older than 10 years old affected by DEB. However, a mean value lower than the reference range was documented in all EB subtypes. Vitamin A levels were normal in most patients. An increase of Vitamin E was observed in patients aged 0–1 years old with DEB (Table 8).

### Nephro-urological complications

Nephro-urological complications occurred in 5.32% of all patients, with DEB patients being the most affected (data not shown). Phimosis was detected in 3 patients (2 DEB and 1 with TKS). Renal failure was found in a total of 3 patients, all affected by DEB (1 patient with acute renal failure and 1 with chronic renal failure). Kidney stones, bladder exstrophy, IgA glomerular nephropathy were described in 3 patients with DEB. Hinman Syndrome was identified in 1 patient with JEB.

### Ocular complications

Ocular complications occurred in 6.2% of the patients. In detail, 8 patients with DEB (5%) had corneal lesions. Strabismus was found in a patient with EBS and ocular synechiae were detected in 1 patient with DEB (data not shown).

### Endocrinological complications

Endocrinological complications occurred in a percentage of 4.14% (data not shown).

169 Osteoporosis, hypothyroidism, Hashimotos’ thyroiditis and type 1 diabetes were described. In detail, osteoporosis was found in 3 patients, 2 patients affected by JEB with LAMA3 deficit and 1 patient with TKS. Hypothyroidism was detected in 2 patients with EBD and COL7A1 mutation. Hashimoto’s thyroiditis and type 1 diabetes were documented in 2 patients with TKS.

### Cardiological complications

A percentage of 3.55% had cardiological complications (data not shown).

Rhythm alterations were detected in 2 patients: bradycardia in a patient affected by EBS and tachycardia in a patient with EBD and COL7A1 mutation. Interatrial defect was found in 2 patients with DEB. Intraventricular defect was found in another patient with DEB. Moreover, acute myocardial infarction was described in a JEB patient.

### Other complications

Depression was documented in a percentage of 1.2% (1 patient with DEB and 1 patient with JEB). Epilepsy was found in 1 patient with JEB (data not shown). Obstructive sleep apnea syndrome was found in 2 EB patients. Cleft lip was detected in 1 EBS patient with KRT5 mutation. Flat foot was observed in an EBS patient. Club foot was found in 2 DEB patients with COL7A1 mutation. Death occurred in a percentage of 10% (16 patients).

## Discussion

### Demographics and genetics

EB is a rare disease with systemic involvement, which requires a complex and multidisciplinary medical approach. A slight female prevalence was found in our population, different from the literature, where a gender prevalence has not been shown given the autosomal inheritance of the disease [11]. We documented a mean age at diagnosis higher than the literature where most patients received a diagnosis of the disease between birth and 12 months compared with 36 months in our sample [12]. This could be secondary to the absence of a maternity service and delivery room in our hospital. Moreover, most of our patients were evaluated immediately after birth locally and nationwide from other health professionals and then addressed to our referral center.

RDEB patients are those with major complications requiring frequent consultations in the reference center. Thus, in our study, we detected a higher prevalence of RDEB than other EB subtypes, in accordance with the literature, where the reported DEB prevalence at birth is 2–6 per million births out of an overall EB prevalence of 8–10 per million births [11]. The most frequent mutations identified in our population through genetic analysis were: KRT14 for EBS, LAMA3 and LAMB3 for JEB and COL7A1 for DEB, in accordance with other reports [4, 12]. Further studies to investigate genotype-phenotype correlation are necessary.

The cases reported belong to the same hospital of the patients recently described by Rossi et al. [7].

However, the number of patients and the genetic data seem different because this study is focused on clinical aspects while the other one on laboratory diagnosis and the period of each study is different.

### Complications

Both cutaneous and extracutaneous complications such as pseudo syndactyly, onychodystrophy, alopecia and ankyloglossia, corneal lesions, sepsis, malnutrition, gastrointestinal problems, were more frequently observed in DEB patients in our sample than in the literature [11, 13]. Sepsis is one of the most frequent extra cutaneous complications in EB patients. This was detected in a small percentage of our population, mainly in patients younger than 1-year-old (data not shown). This could be related to the multidisciplinary management of our third level referral hospital for EB patients where paediatricians, dermatologists, haematologists and EB expert nurses and clinicians work together preventing and managing EB patients’ complications. In accordance with the literature, the most frequent pathogen found in EB infections was *S.aureus* [14]. In more detail, skin infections were described in 5.9% of our population, 210 with DEB patients being the most affected (69%). Indeed, the COL7A1 mutation detected in DEB patients has been related to chronic wounds, bacterial colonization, skin infections and skin cancer [15–19]. While sepsis proved to be the major cause of morbidity and mortality in younger patients, squamous cell carcinoma was mostly observed in older patients, most of whom were affected by DEB, in accordance with literature findings [20]. Monitoring of chronic wounds is a key element in EB patients in order to promptly diagnose skin cancer [14]. Indeed, the relation between skin damage and skin cancer is well known: chronic wounds lead to aberrant stimulation of inflammation, fibrosis and tumour progression [15, 21]. Moreover, specific gene mutations identified in EB patients are responsible for altering the healing process, thus favouring epithelial cancers growth [20]. In our sample, death occurred because of severe EB with skin cancers and metastasis in a percentage of 10% (16 patients). The primary health care evaluation of EB patients should focus on wound healing changes in order to promptly diagnose tumour occurrence.

Other common problems affecting EB patients involved the gastrointestinal system and nutritional aspects. Among these complications, esophageal stenosis, constipation, dental caries, and malnutrition were the most frequently reported. A percentage as high as 81.4% of DEB patients underwent esophageal dilatation (data not shown), in accordance with literature reports [13]. Frequently, more than one dilatation per patient was required because of numerous relapses. In our series, 35.6% of patients with DEB underwent more than one esophageal dilatation. Constipation was found in 21.3% of patients, especially those with DEB, in line with literature findings [13]. This complication is secondary to anus lesions related to low-fibre diet, poor fluid intake, delayed defecation, and exacerbated by oral iron supplementation for anemia.

According to literature, GERD prevalence may be underestimated as its assessment is often based on patient recall [22].Moreover, further studies should be performed in order to better identify and manage this problem.

### Anemia, nutrition and growth

Anemia represents one of the most important complications of DEB patients [23 ex 21], as also shown by our findings (66%). Although hemoglobin was found to be below normal ranges for age, the mean ferritin value found was 81.5 ng/mL (normal range: 9-290 ng/ml), with values sometimes above the normal limit (highest observed value: 1039 ng/ml), reflecting the chronic inflammation typical of EB patients. Therefore, regular serum iron dosing could be particularly useful in these patients. Indeed, mean serum iron values below the normal range limit (35-130 mcg/dl) were observed in patients older than 20 years (27.6 mcg/dl). In this context, adequate evaluation of anemia should include a complete iron asset examination with serum iron values. Oral iron supplementation is widely used to treat anemia even if it is poorly tolerated and associated with gastrointestinal disorders such as: heartburn, constipation or diarrhea, with a consequent reduction in compliance [24]. Moreover, the reduced gastrointestinal absorption, chronic losses, nutritional deficiencies and chronic inflammatory state, can lead to transfusion-dependent patterns 241 in EB patients [25]. Further research is necessary to study iron formulations to lengthen the time to transfusion and improve the compliance of children with EB. While the role of vitamin D in bone functions, immunomodulatory and anti-inflammatory actions is well-known [26, 27], only a few studies described the role of Vitamin D deficiency as a contributor to anemia [27]. In our sample, we described a vitamin D mean value lower than the reference in all EB subtypes. It is widely known that gastrointestinal malabsorption, lack of sun exposure, inadequate nutrition can all interfere with both vitamin D serum levels and adequate peak bone mass [28], along with limited physical activity and chronic inflammation [28]. According to the literature, children with DEB and JEB have a lower BMD than subjects with EBS [29]. Nevertheless, only 3 patients of our population presented osteoporosis. It should be noted that our EB patients are constantly monitored with dual energy X-ray after puberty, while receiving specific vitamin supplementations and dietetic advice for a variegated and balanced nutrition. Although dual energy X-ray is a second level examination performed in specialised centers, dietetic advice and vitamin supplementation should be considered by primary care physicians in the management of EB. In order to treat anemia, nutrition needs to be addressed first in EB patients. Malnutrition and vitamin deficiencies have been related to growth delay and anemia in those patients [30]. In particular, researchers found that low levels of hemoglobin, iron, vitamin D, albumin and high levels of C-reactive protein relate to low weight in EB patients [30]. In our study, we demonstrated a strong association of low serum albumin and feeding problems with a low BMI centile. In our series, malnutrition was observed in more than a third of patients, especially in those affected by DEB, with a low BMI adjusted for age. Moreover, malnutrition was more common in patients aged 1-10 years and older than 20 years. In particular, some Researchers described initial weight gain of JEB as an important prognostic factors predicting both death and skin carcinoma [31, 32].

None of our patients performed tube feeding because our methodology is to perform a prompt diagnosis and adequate treatment of esophageal stenosis in addition with nutritional support.

Moreover, patients require higher protein because of protein loss through skin lesions and inflammatory processes, as discussed in literature [33].

In particular, according to nutritional experts, protein intake should be up to 200% of the recommended intake [34].

To better define EB patient’s growth, specific growth charts have been recently proposed [30]. For example, researchers found out that the predicted median weight in EB patients aged 20 years old should be 35.2 kg for male and 40.1 kg for female [30]. A specific diet with a balance of all macronutrients is required, but this should take into account personal tastes, poor dentition, food aversion and children behaviour, such as apathy, which has been frequently described [24, 25]. Moreover, improving caloric intake with sugar limitations is fundamental: sucrose contained in foods and analgesic must be avoided because it has been associated with dental caries and tooth decay, which are frequently observed in EB patients both in the literature and in our population [24]. Dental problems are in turn responsible for a decreased intake of micronutrients such as vitamins and minerals [24]. All pediatricians should consider EB patient’s nutritional requirements while collaborating with other health professionals involved such as dentists [35].

In contrast to literature findings, we found out that the majority of our patients had 271 normal values of vitamin A and high levels of vitamin E. These findings are rarely observed in both the general population and EB patients. Our findings could be probably explained by the overuse of topic agents containing vitamin E in this population, where the lack of an adequate hydrolipidic film is connected to the absence of skin protective function and to a more percutaneous absorption. Moreover, the general nutritional deficit detected in our cohort demonstrates that nutritional supplementation should always be recommended as the risk of over dosage is low [30]. As reported in literature, chronic inflammation, caused by wounds and bacterial skin infections, have been related to malnutrition [30]. In particular, CRP, leukocytes and immunoglobulins increments were detected in our population as were in literature [30, 36, 37]. In patients younger than 20 years old, which are less affected by malnutrition, lower levels of inflammation were documented. In contrast, patients older than 20 years old, where higher levels of CRP and immunoglobulins were found, also proved to be the most affected by malnutrition. These results could confirm the relationship between chronic inflammation and malnutrition. Indeed, anecdotal use of anti-inflammatory drugs has been proposed in order to improve both skin wounds and anemia [30].

Reports on quality of life (QoL) of EB patients show the heavy burden of this disease on families, which requires psychological individualised support and close monitoring following psychological changes and specific physical requirements [38, 39]. Specifical questionnaire have been purposed to assess QoL of EB patients [40].

In our population, a low rate of depression was detected, probably underestimating the real psychological consequences of the disease. Further studies are necessary to evaluate EB patient’s QoL, thus helping paediatricians-dermatologists cooperation and the multidisciplinary equipe to manage with psychological consequences of EB for both patients and families. Moreover, pediatricians should refer EB patients and families to patients’ associations in addition with social workers which play an important role in counselling families of affected children.

### Monitoring

Laboratory, imaging and clinical monitoring are important aspects of EB management. Patients with EB require regular monitoring for complications and sequelae. The frequency of the evaluations varies depending on age and EB subtypes. All EB patients should perform vaccinations as recommended in the specific Country Vaccines Plan. Tables 7 and 9 summarize our recommendations for the management of children with EB.

**Table 9:** Recommendations on EB patient’s management for pediatricians

Nutritional care Soft diet Oral intake 150–200% Vitamin supplementation, iron supplementation Gastrostomy, if necessary 6-12 months growth check	Gastroenterological care Laxatives Endoscopist evaluation Esophageal dilatation
**Dental care** Delicate oral hygiene Avoiding sucrose Dental prevention with programmed check	**Emotional care** Psychological support for both patient and families/caregivers Pain monitoring and treatment (NSAIDS and acetaminophen, tramadol, opioids, gabapentin) Antihistamines (with eventually antidepressants and oral gabapentin or pregabalin) for chronic pruritus Topical anesthetic for oral pain
**Ocular care** Lubricants Topical antibiotics in case of corneal erosions Orthoptic surveillance for refractive errors and strabismus Regular check	**Mobility care** Physical specialists for mobility support Footwear specific for EB patients Plastic surgery in case of pseudo syndactyly in case of compromission of patients’ independence Occupational therapy
**Skin care** Advanced dressings, emollients, frictions avoidance Skin infections treatment: bleach baths or compresses, topical antiseptics, and topical antibiotics. Bacterial cultures of critically colonized wounds are not routinely performed. If required, cultures should be obtained before starting topical antimicrobial treatment Topical antiseptic agents include chlorhexidine, benzalkonium chloride, and silver sulfadiazine (small areas for short period because of side effects connected to systemic silver toxicity) Topical antibiotics (mupirocin, fusidic acid) should be used with caution to avoid the antibiotic-resistant bacteria. If used, a rotation of different agents is recommended every two to six weeks to minimize the induction of bacterial resistance In case of severe wound infections, systemic antibiotics based on antibiogram results Regular assessment of skin lesions and multiple biopsies of chronic wounds in order to promptly diagnose skin cancer	

## Conclusions

EB is a rare disease with a wide range of clinical presentations, from mild 300 to devastating severe forms. Diagnosis and management are both challenging: specialised hospitals should be the referral centre for this complex role guaranteeing a multidisciplinary evaluation and follow-up. However, the role of the family pediatrician is regularly connected with the reference center in order to reduce difficulties related to the management and to prevent/delay severe complications.

Considering EB rarity, the family pediatrician should be informed about EB peculiarity and specifically skin fragility. However, the pediatrician should be involved in the follow-up of EB patients reducing the frequency of moving to the reference center.

This study highlights the most frequent complications in EB severe subtypes focusing on nutritional and gastrointestinal aspects. Multidisciplinary care involving pediatrician-dermatologist cooperation within specialist clinicians, families and patients Association is fundamental, providing instruments to face the disease and improve EB QoL.

## Supplementary Information

**Additional file 1.** **Supplementary Table 1.** Laboratory workup of EBS patients per age group. **Supplementary Table 2.** Laboratory workup of JEB patients per age group.**Supplementary Table 3.** Laboratory workup of DEB patients per age group.

## Data Availability

At Bambino Gesù Children Hospital, Dr. Marchili’s repository.

## Abbreviations

EBS: Epidermolysis bullosa
EBD: Dystrophic epidermolysis bullosa
JEB: Junctional epidermolysis bullosa
QoL: Quality of Life
