# Supplementary material for: Epidermolysis Bullosa in children: the central role of the pediatrician
Source: Orphanet J Rare Dis. 2022 Apr 4;17:147. doi: 10.1186/s13023-021-02144-1 (PMC8978425; doi:10.1186/s13023-021-02144-1)
Supplement: Supplementary file 1 — Additional file 1. Supplementary Table 1. Laboratory workup of EBS patients per age group. Supplementary Table 2. Laboratory workup of JEB patients per age group.Supplementary Table 3. Laboratory workup of DEB patients per age group. [file 13023_2021_2144_MOESM1_ESM.docx]

| Supplementary data. Table 1. Laboratory workup of EBS patients per age group. | | | | | |
| --- | --- | --- | --- | --- | --- |
|  | **0-1 yr** | **1-10 yr** | **10-20 yr** | **>20 yr** |  |
| *Anemia* |  |  |  |  |  |
| Haemoblobin (g/dl) | 12.4 (9.2 – 13.5) ± 1.22 | 12.6 (12.1 – 14.9) ± 1.49 | N/A | N/A |  |
| Reticulocytes (%) | N/A | 0.48 (0.40 – 0.57) ± 0.12 | N/A | N/A |  |
| Serum iron (mcg/dl) | 58.5 (18 – 109) ± 28.7 | 47 (32 – 62) ± 21.2 | N/A | N/A |  |
| Ferritin (ng/ml) | 29 (18 – 31) ± 5.3 | 46 (18-74) ± 39.6 | N/A | N/A |  |
| Transferrin (mg/dl) | 271 (244 – 302) ± 25 | 286 (257 – 315) ± 41 | N/A | N/A |  |
| *Inflammation* |  |  |  |  |  |
| White blood cells (cells/mm3 *10^3^) | 8.82 (4.54 – 11.83) ± 2.74 | 9.39 (9.23 – 9.55) ± 0.22 | N/A | N/A |  |
| Neutrophils (%) | 42.4 (34.6 – 56) ± 8.76 | 60.6 (48.7 – 72.5) ± 16.8 | N/A | N/A |  |
| CRP (mg/dl) | 0.15 (0.05 – 0.21) ± 0.08 | 1.55 (0.05 – 3.05) ± 2.1 | N/A | N/A |  |
| ESR (mm/h) | 8 (6 – 26) ± 8.3 | N/A | N/A | N/A |  |
| IgA (mg/dl) | 166 (66 – 214) ± 71.8 | N/A | N/A | N/A |  |
| IgG (mg/dl) | 1013 (731 – 1550) ± 344 | N/A | N/A | N/A |  |
| IgM (mg/dl) | 81 (42 – 128) ± 35 | N/A | N/A | N/A |  |
| *Nutritional state* |  |  |  |  |  |
| Glicemia (mg/dl) | 81 (79 – 98) ± 7.9 | 82 (78 – 85) ± 4.9 | N/A | N/A |  |
| Blood protein (g/dl) | 6.7 (6.2 – 8) ± 0.8 | 7 (6.8 – 7.2) ± 0.3 | N/A | N/A |  |
| Albumin (g/dl) | 4.4 (4.2 – 4.7) ± 0.2 | 4.4 (4.1 – 4.6) ± 0.4 | N/A | N/A |  |
| Pre-albumin (mg/dl) | 17 (15 – 17) ± 1.2 | N/A | N/A | N/A |  |
| RBP (mg/dl) | 2.3 (1.7 – 2.6) ± 0.5 | N/A | N/A | N/A |  |
| Total cholesterol (mg/dl) | 137 (119 – 182) ± 24 | 163 (147 – 180) ± 23.3 | N/A | N/A |  |
| Triglycerides (mg/dl) | 43 (40 – 73) ± 13 | 55 (49 – 61) ± 8.5 | N/A | N/A |  |
| Vitamin A (μmol/l) | 1.3 (1.22 – 1.94) ±0.39 | N/A | N/A | N/A |  |
| Vitamin D (ng/ml) | 25 (19 – 31.1) ± 5.8 | N/A | N/A | N/A |  |
| Vitamin E (μmol/l) | 51.2 (33.3 – 53.9) ± 11.2 | N/A | N/A | N/A |  |
| Vitamin B12 (pg/ml) | 632 (452 – 1225) ± 404 | N/A | N/A | N/A |  |
| Folati (ng/ml) | 6 (4 – 7.2) ± 1.6 | N/A | N/A | N/A |  |
| Vitamin B6 (μmol/l) | 60.5 (41.1 – 69.8) ± 14.6 | N/A | N/A | N/A |  |
| *Renal function* |  |  |  |  |  |
| Azotemia (mg/dl) | 14 (12 -14) ± 1 | 15 (13 – 17) ± 3 | N/A | N/A |  |
| Creatinine (mg/dl) | 0.47 (0.22 – 0.54) ± 0.14 | 0.53 (0.35 – 0.7) ± 0.25 | N/A | N/A |  |
| Uric acid (mg/dl) | 3.2 (2.5 – 3.9) ± 0.59 | 3.85 (3.8 – 3.9) ±0.07 | N/A | N/A |  |
| *Liver function* |  |  |  |  |  |
| AST (IU/l) | 32 (26- 39) ± 5.3 | 26 (24 – 27) ± 2.1 | N/A | N/A |  |
| ALT (IU/l) | 19 (13 – 25) ± 8.5 | N/A | N/A | N/A |  |
| GGT (IU/l) | 8.5 (6 – 9) ± 1.4 | 11 (8 -14) ± 4.24 | N/A | N/A |  |
| Bilirubin (mg/dl) | 0.4 (0.3 – 0.5) ± 0.1 | N/A | N/A | N/A |  |
| Alkaline phosphatase (U/L) | 398 (381 – 454) ± 38 | 436 (428 – 444) ± 11 | N/A | N/A |  |
| *Coagulation* |  |  |  |  |  |
| Platelets (cells/mm3*10^3^) | 285 (208 – 327) ± 43 | 240 (202 – 278) ± 54 | N/A | N/A |  |
| Fibrinogen (mg/dl) | 303 (239 – 366) ± 64 | N/A | N/A | N/A |  |
| PT (s) | 13.3 (12.4 – 13.5) ± 0.59 | N/A | N/A | N/A |  |
| aPTT (s) | 31.3 (26.8 – 32.0) ± 2.82 | N/A | N/A | N/A |  |
| *Electrolytes* |  |  |  |  |  |
| Sodium (mmol/l) | 139 (136 – 141) ± 2.1 | 142 (140 – 143) ± 2.1 | N/A | N/A |  |
| Potassium (mmol/l) | 4.6 (4.1 – 4.9) ± 0.4 | 4.6 (4.57 – 4.6) ± 0.02 | N/A | N/A |  |
| Chlorine (mmol/l) | 104 (101 – 108) ± 3.3 | 106 (105 – 106) ± 0.7 | N/A | N/A |  |
| Calcium (mg/dl) | 9.5 (9.2 – 9.9) ± 0.3 | 9.9 (9.8 – 10) ± 0.1 | N/A | N/A |  |
| Magnesium (mg/dl) | 2.1 (2.0 – 2.2) ± 0.08 | N/A | N/A | N/A |  |
| Phosphate (mg/dl) | 5.1 (4.5 – 5.3) ± 0.3 | 4.8 (4.4 – 5.3) ±0.6 | N/A | N/A |  |
| *Hormones* |  |  |  |  |  |
| PTH (pg/ml) | 25 (21 – 29) ± 4 | N/A | N/A | N/A |  |
| TSH (U/ml) | 2.85 (2.51 – 3.84) ± 0.59 | N/A | N/A | N/A |  |
| FT4 (ng/dl) | 1.16 (1.07 – 1.23) ± 0.08 | N/A | N/A | N/A |  |
| *Other* |  |  |  |  |  |
| Amylase (U/L) | 66 (56 – 96) ± 21 | N/A | N/A | N/A |  |
| Copper (mcr/dl) | 82 (79 – 95) ± 8.3 | N/A | N/A | N/A |  |

| Supplementary data. Table 2. Laboratory workup of JEB patients per age group. | | | | |
| --- | --- | --- | --- | --- |
|  | **0-1 yr** | **1-10 yr** | **10-20 yr** | **>20 yr** |
| *Anemia* |  |  |  |  |
| Haemoblobin (g/dl) | 10.8 (5.5 – 17.4) ± 3.9 | N/A | N/A | 10.9 (9.5 – 13.5) ± 2.0 |
| Reticulocytes (%) | 1.9 (1.3 – 2.5) ± 0.9 | N/A | N/A | 1.4 (0.8 – 1.5) ± 0.4 |
| Serum iron (mcg/dl) | 44 (12 – 48) ± 19.7 | N/A | N/A | 41 (13- 45) ± 17.4 |
| Ferritin (ng/ml) | 13.5 (8 – 19) ± 7.8 | N/A | N/A | 14.0 (11 – 48) ± 20.5 |
| Transferrin (mg/dl) | N/A | N/A | N/A | 238 (215 – 287) ± 36.8 |
| *Inflammation* |  |  |  |  |
| White blood cells (cells/mm3 *10^3^) | 11.5 (9.1 – 13.8) ± 3.3 | N/A | N/A | 10.2 (8.7 – 11.9) ± 1.6 |
| Neutrophils (%) | 57.5 (51.2 – 63.9) ± 9.0 | N/A | N/A | 65.3 (54.1 – 65.9) ± 6.6 |
| CRP (mg/dl) | N/A | N/A | N/A | 2.13 (2.03 – 4.15) ± 1.19 |
| ESR (mm/h) | N/A | N/A | N/A | 35 (14 – 119) ± 56 |
| IgA (mg/dl) | 223 (156 – 290) ± 94.8 | N/A | N/A | 632 (431 – 988) ± 282 |
| IgG (mg/dl) | 1441 (1132 – 1751) ± 438 | N/A | N/A | 1313 (1104 – 1766) ± 338 |
| IgM (mg/dl) | 110 (93 – 127) ± 24 | N/A | N/A | 87 (70 – 96) ± 13 |
| *Nutritional state* |  |  |  |  |
| Glicemia (mg/dl) | 72.5 (57 – 88) ± 21.9 | N/A | N/A | 95 (83 – 97) ± 7.6 |
| Blood protein (g/dl) | 7.25 (7.2 – 7.3) ± 0.07 | N/A | N/A | 7.6 (7 – 7.7) ± 0.38 |
| Albumin (g/dl) | 4 (3.7 – 4.3) ± 0.42 | N/A | N/A | 4.1 (3.4 – 4.2) ± 0.44 |
| Pre-albumin (mg/dl) | N/A | N/A | N/A | 17 (15 – 23) ± 4.2 |
| RBP (mg/dl) | N/A | N/A | N/A | 3.6 (2.39 – 3.6) ± 0.69 |
| Total cholesterol (mg/dl) | 139 (122 – 156) ± 24 | N/A | N/A | 169 (168 – 187) ± 10.7 |
| Triglycerides (mg/dl) | 98.5 (80 – 117) ± 26 | N/A | N/A | 79 (79 – 115) ± 20.8 |
| Vitamin A (μmol/l) | N/A | N/A | N/A | 1.3 (1 – 3.9) ± 1.6 |
| Vitamin D (ng/ml) | 18.8 (10.8 – 26.8) ± 11.3 | N/A | N/A | 19.5 (14 – 27.2) ± 6.6 |
| Vitamin E (μmol/l) | N/A | N/A | N/A | 36.6 (29.2 – 100.5) ± 39.2 |
| Vitamin B12 (pg/ml) | N/A | N/A | N/A | 457 (261 – 545) ± 145 |
| Folati (ng/ml) | N/A | N/A | N/A | 7.4 (6 – 20.9) ± 8.3 |
| Vitamin B6 (μmol/l) | N/A | N/A | N/A | 15 (15 – 31) ± 9.2 |
| *Renal function* |  |  |  |  |
| Azotemia (mg/dl) | 11.5 (11 – 12) ± 0.7 | N/A | N/A | 24 (12 – 33) ± 10.5 |
| Creatinine (mg/dl) | 0.29 (0.19 – 0.39) ± 0.14 | N/A | N/A | 0.63 (0.62 – 0.70) ± 0.04 |
| Uric acid (mg/dl) | 4.0 (3.9 – 4.09) ± 0.07 | N/A | N/A | 5.6 (4.2 – 19) ± 8.2 |
| *Liver function* |  |  |  |  |
| AST (IU/l) | 28.5 (24 – 33) ± 6.4 | N/A | N/A | 16 (14 – 26) ± 6.4 |
| ALT (IU/l) | 16.5 (8 – 25) ± 12 | N/A | N/A | 10 (9 – 22) ± 7.2 |
| GGT (IU/l) | N/A | N/A | N/A | 12 (11 – 14) ± 1.5 |
| Bilirubin (mg/dl) | 0.33 (0.19 – 0.46) ± 0.19 | N/A | N/A | 0.4 (0.3 – 1.27) ± 0.53 |
| Alkaline phosphatase (U/L) | 353 (209 – 497) ± 204 | N/A | N/A | 67 (61 – 171) ± 62 |
| *Coagulation* |  |  |  |  |
| Platelets (cells/mm3*10^3^) | 364 (241 – 486) ± 173 | N/A | N/A | 291 (257 – 342) ± 42.8 |
| Fibrinogen (mg/dl) | N/A | N/A | N/A | 462 (368 – 511) ± 72.7 |
| PT (s) | N/A | N/A | N/A | 10 (10 – 12.9) ± 1.67 |
| aPTT (s) | N/A | N/A | N/A | 29.8 (28.9 – 30.3) ± 0.71 |
| *Electrolytes* |  |  |  |  |
| Sodium (mmol/l) | 137.5 (136 – 139) ± 2.1 | N/A | N/A | 140 (139 – 141) ± 1 |
| Potassium (mmol/l) | 4.58 (4.55 – 4.61) ± 0.04 | N/A | N/A | 4.40 (4.23 – 4.68) ± 0.23 |
| Chlorine (mmol/l) | 103 (100 – 106) ± 4.2 | N/A | N/A | N/A |
| Calcium (mg/dl) | 9.6 (9.5 – 9.7) ± 0.14 | N/A | N/A | 8.9 (8.8 – 9.3) ± 0.26 |
| Magnesium (mg/dl) | 1.99 (1.98 – 2.01) ± 0.02 | N/A | N/A | 2.0 (1.85 – 2.07) ± 0.11 |
| Phosphate (mg/dl) | 5.2 (5 – 5.4) ± 0.28 | N/A | N/A | 3.4 (3.3 – 3.6) ± 0.15 |
| *Hormones* |  |  |  |  |
| PTH (pg/ml) | N/A | N/A | N/A | 54 (36 – 63) ± 13.7 |
| TSH (U/ml) | N/A | N/A | N/A | 1.22 (0.91 – 2.3) ± 0.73 |
| FT4 (ng/dl) | N/A | N/A | N/A | 1.31 (1.22 – 2.69) ± 0.82 |
| *Other* |  |  |  |  |
| Amylase (U/L) | 56 (30 – 82) ± 36.8 | N/A | N/A | 55 (44 – 139) ± 52 |
| Copper (mcr/dl) | N/A | N/A | N/A | 103 (90 – 169) ± 42 |

| Supplementary data. Table 3. Laboratory workup of DEB patients per age group. | | | | |
| --- | --- | --- | --- | --- |
|  | **0-1 yr** | **1-10 yr** | **10-20 yr** | **>20 yr** |
| *Anemia* |  |  |  |  |
| Haemoblobin (g/dl) | 10.3 (6.9 – 12.8) ± 16.9 | 10.7 (7.8 – 13.5) ± 1.8 | 8.9 (5.3 – 13) ± 2.5 | 10.3 (7.6 – 14.3) ± 1.9 |
| Reticulocytes (%) | 1.7 (0.8 – 12.8) ± 4.3 | 1.6 (1.3 – 2.2) ± 0.3 | 1.6 (1.1 – 2.1) ± 0.3 | 1.7 (0.9 – 2.5) ± 0.6 |
| Serum iron (mcg/dl) | 22 (10 – 477) ± 99 | 19 (10 – 62) ± 19 | 26 (5 -112) ± 34 | 15 (11 – 98) ± 26 |
| Ferritin (ng/ml) | 21 (4 – 428) ± 88 | 11 (6 – 135) ± 44 | 26 (6 -1039) ± 378 | 32 (4 – 843) ± 284 |
| Transferrin (mg/dl) | 252 (97 – 421) ± 75 | 285 (233 – 347) ± 52 | 182 (92 – 358) ± 82 | 198 (147 – 302) ± 46 |
| *Inflammation* |  |  |  |  |
| White blood cells (cells/mm3 *10^3^) | 10.2 (4.57 – 16.67) ± 3.48 | 10.87 (7.61 – 13.99) ± 2.11 | 9.41 (3.95 – 13.03) ± 3.07 | 8.86 (6 – 15.06) ± 2.71 |
| Neutrophils (%) | 50.8 (8.7 – 81.3) ± 16.07 | 58.3 (6.9 – 67) ± 18.7 | 59.9 (68.2 – 66.7) ± 27.5 | 63.4 (53.7 – 77.9) ± 7.16 |
| CRP (mg/dl) | 2.94 (0.05 – 15.81) ± 4.44 | 2.31 (0.24 – 6.28) ± 2.17 | 10.31 (0.05 – 14.3) ± 5.7 | 3.52 (0.85 – 10.84) ± 2.86 |
| ESR (mm/h) | 27.5 (5 – 74) ± 24.5 | 29 (8 – 65) ± 21.6 | 48 (20 – 58) ± 17.6 | 61 (9 – 90) ± 41 |
| IgA (mg/dl) | 263 (5 – 525) ± 183 | 204 (99 – 643) ± 205 | 414 (225 – 831) ± 263 | 417 (173 – 809) ± 198 |
| IgG (mg/dl) | 2222 (616 – 6564) ± 1562 | 1795 (1100 – 3013) ± 638 | 1868 (1163 – 5788) ± 2041 | 2700 (1379 – 5952) ± 1493 |
| IgM (mg/dl) | 104 (51 – 198) ± 39.8 | 133 (46 – 357) ± 97 | 144 (37 – 247) ± 64.3 | 141 (90 – 491) ± 129 |
| *Nutritional state* |  |  |  |  |
| Glicemia (mg/dl) | 84 (70 – 108) ± 9 | 87 (74 – 104) ± 10.7 | 94 (68 – 107) ± 15.3 | 82 (69 – 107) ± 10.8 |
| Blood protein (g/dl) | 7.4 (6.4 – 10.8) ± 1.03 | 8 (6.5 – 8.8) ± 0.88 | 7.7 (6.8 – 10.6) ± 1.21 | 7.7 (7.2 – 10) ± 0.86 |
| Albumin (g/dl) | 3.8 (2.5 – 4.5) ± 0.53 | 4 (3.3 – 4.6) ± 0.44 | 3.4 (2.07 – 4.2) ± 0.84 | 3.8 (3.4 – 4.5) ± 0.38 |
| Pre-albumin (mg/dl) | 13 (6 – 20) ± 3.9 | 12 (1.1 – 18) ± 6.2 | 12 (6 – 18) ± 5.5 | 10 (9 – 17) ± 3.1 |
| RBP (mg/dl) | 1.91 (1.17 – 2.86) ± 5.2 | 1.58 (1.45 – 2.56) ± 0.42 | 2.24 (1.61 – 2.57) ± 0.35 | 1.95 (1.63 – 2.88) ± 0.45 |
| Total cholesterol (mg/dl) | 135 (116 – 167) ± 15.5 | 139 (96 – 173) ± 25.7 | 103 (92 – 124) ± 14.1 | 127 (67 – 216) ± 41.3 |
| Triglycerides (mg/dl) | 66 (49 – 156) ± 29.7 | 84.5 (43 – 167) ± 39.8 | 60 (41 – 235) ± 79.9 | 82 (45 – 98) ± 19.5 |
| Vitamin A (μmol/l) | 1.1 (0.5 – 2.72) ± 0.79 | 0.94 (0.7 – 1.2) ± 0.22 | 1.22 (0.4 – 1.94) ± 0.64 | 1.19 (0.94 – 2.3) ± 0.46 |
| Vitamin D (ng/ml) | 13 (4 – 39) ± 11 | 22 (4 – 29) ± 8.6 | 6.9 (6 – 60) ± 27 | 12 (5 – 42) ± 13 |
| Vitamin E (μmol/l) | 57 (25 – 105) ± 27 | 48 (26 – 67) ± 16 | 50 (31 – 69) ± 17 | 68 (25 – 90) ± 20 |
| Vitamin B12 (pg/ml) | 710 (445 – 1398) ± 316 | 681 (447 – 1586) ± 403 | 431 (349 – 1003) ± 286 | 408 (173 – 791) ± 200 |
| Folati (ng/ml) | 8 (2.8 – 58) ± 15 | 7 (4.4 – 50) ± 16.8 | 10 (4 – 15.8) ± 5 | 6.5 (2.2 – 47) ± 18 |
| Vitamin B6 (μmol/l) | 41 (17 – 212) ± 71 | 55 (30 – 141) ± 42.7 | 20 (10 – 44) ± 15 | 34 (20 – 56) ± 15 |
| *Renal function* |  |  |  |  |
| Azotemia (mg/dl) | 9 (4 – 15) ± 3 | 8 (6 – 19) ± 4.4 | 10 (4 -27) ± 7.6 | 13.5 (10 – 20) ± 3.3 |
| Creatinine (mg/dl) | 0.31 (0.15 – 0.73) ± 0.12 | 0.34 (0.16 – 0.46) ± 0.10 | 0.61 (0.26 – 1.28) ± 0.34 | 0.62 (0.28 – 0.78) ± 0.17 |
| Uric acid (mg/dl) | 3.1 (1.2 – 4.5) ± 0.7 | 3.4 (2.4 – 6.5) ± 1.3 | 4.2 (2.9 – 11) ± 3.1 | 4.6 (1.9 – 4.9) ± 0.87 |
| *Liver function* |  |  |  |  |
| AST (IU/l) | 25 (11 – 90) ± 16.4 | 29 (10 – 34) ± 7.9 | 17 (15 – 28) ± 4.8 | 19 (13 -24) ± 3.6 |
| ALT (IU/l) | 10 (5 – 46) ± 8.6 | 10 (6 – 32) ± 7.9 | 13 (8 – 34) ± 9 | 10 (7 – 20) ± 3.9 |
| GGT (IU/l) | 8 (5 – 915) ± 203 | 7 (5 – 14) ± 3.2 | 14 (4 – 87) ± 31 | 14 (6 – 21) ± 5.4 |
| Bilirubin (mg/dl) | 0.18 (0.14 – 0.58) ± 0.12 | 0.25 (0.18 – 0.4) ± 0.73 | 0.35 (0.16 – 0.55) ± 0.14 | 0.35 (0.14 – 0.74) ± 0.2 |
| Alkaline phosphatase (U/L) | 328 (98 – 639) ± 162 | 399 (201 – 573) ± 137 | 203 (146 – 345) ± 92 | 206 (128 – 289) ± 51 |
| *Coagulation* |  |  |  |  |
| Platelets (cells/mm3*10^3^) | 486 (55 – 1183) ± 232 | 515 (274 – 828) ± 149 | 412 (211 – 697) ± 141 | 326 (250 – 509) ± 83 |
| Fibrinogen (mg/dl) | 427 (303 – 669) ± 107 | 481 (393 – 550) ± 60 | 486 (275 – 693) ± 164 | 500 (372 – 731) ± 118 |
| PT (s) | 14 (12 – 16) ± 1.1 | 14 (13 – 15) ± 0.6 | 15 (13 – 18) ± 1.8 | 14 (12 – 17) ± 1.3 |
| aPTT (s) | 35 (28 – 40) ± 3.6 | 34 (28 – 42) ± 5.4 | 36 (29 – 46) ± 6.9 | 35 (30 – 40) ± 3.3 |
| *Electrolytes* |  |  |  |  |
| Sodium (mmol/l) | 138 (130 – 142) ± 2.8 | 139 (137 – 141) ± 1.4 | 137 (131 – 142) ± 4.1 | 139 (135 – 143) ± 2.3 |
| Potassium (mmol/l) | 4.6 (3.77 – 5.4) ± 0.42 | 4.1 (3.83 – 4.82) ± 0.33 | 4.1 (3.5 – 4.9) ± 0.44 | 4.29 (3.89 – 4.66) ± 0.25 |
| Chlorine (mmol/l) | 102 (98 – 111) ± 3.2 | 104 (100 – 106) ± 2.2 | 105 (102 – 107) ± 2.3 | 104 (101 – 109) ± 2.5 |
| Calcium (mg/dl) | 9.3 (8.2 – 10) ± 0.4 | 9.5 (8.5 – 9.9) ± 0.44 | 8.95 (2.08 – 9.6) ± 2.85 | 8.9 (8.5 – 9.9) ± 0.42 |
| Magnesium (mg/dl) | 2.17 (1.71 – 2.5) ± 0.2 | 2.07 (1.9 – 2.29) ± 0.13 | 1.99 (0.75 – 2.1) ± 0.52 | 2.09 (1.65 – 3.94) ± 0.62 |
| Phosphate (mg/dl) | 4.9 (3.5 – 6.3) ± 0.71 | 4.65 (3.8 – 5.3) ± 0.47 | 3.9 (1.17 – 4.7) ± 1.29 | 4.19 (3.2 – 4.6) ± 0.51 |
| *Hormones* |  |  |  |  |
| PTH (pg/ml) | 30 (9 – 59) ± 14 | 21 (8 – 129) ± 46 | 40 (14 – 1285) ± 511 | 34.5 (22 – 83) ± 25 |
| TSH (U/ml) | 1.83 (0.72 – 5.12) ± 1.33 | 2.24 (0.84 – 4.98) ± 1.36 | 2.83 (1.01 – 4.03) ± 1.01 | 1.64 (0.76 – 4.56) ± 1.33 |
| FT4 (ng/dl) | 1.17 (0.91 – 1.5) ± 0.17 | 1.36 (0.8 – 1.66) ± 0.26 | 1.01 (0.79 – 1.12) ± 1.21 | 1 (0.8 – 1.25) ± 1.14 |
| *Other* |  |  |  |  |
| Amylase (U/L) | 64 (24 – 189) ± 39.6 | 57.5 (39 – 103) ± 22.5 | 53.5 (27 – 126) ± 42.6 | 99 (72 – 670) ± 235.5 |
| Copper (mcr/dl) | 111 (72 – 156) ± 25 | 119 (9.7 – 140) ± 48 | 71 (55 – 87) ± 23 | 98 (60 – 121) ± 26 |
